# Transferable Plasmid-Borne *mcr-1* in a Colistin-Resistant Shigella flexneri Isolate

**DOI:** 10.1128/AEM.02655-17

**Published:** 2018-04-02

**Authors:** Beibei Liang, Adam P. Roberts, Xuebin Xu, Chaojie Yang, Xiaoxia Yang, Jinyan Wang, Shengjie Yi, Yongrui Li, Qiuxia Ma, Fuli Wu, Shaofu Qiu, Hongbin Song

**Affiliations:** a Academy of Military Medical Sciences, Academy of Military Sciences, PLA, Beijing, China; b Institute of Disease Control and Prevention, PLA, Beijing, China; c Department of Parasitology, Liverpool School of Tropical Medicine, Liverpool, United Kingdom; d Research Centre for Drugs and Diagnostics, Liverpool School of Tropical Medicine, Liverpool, United Kingdom; e Shanghai Municipal Center for Disease Control and Prevention, Shanghai, China; f Beijing igeneCode Biotech Co., Ltd., Beijing, China; INRS—Institut Armand-Frappier

**Keywords:** IS*Apl1*, Tn*6390*, multidrug resistance, plasmid transfer, composite transposon

## Abstract

Since the initial discovery of *mcr-1* in an Escherichia coli isolate from China, the gene has also been detected in Klebsiella pneumoniae and Salmonella enterica but is rarely reported in other Enterobacteriaceae. Here, we report the isolation and identification of a Shigella flexneri strain harboring *mcr-1* from stool samples in a pig farm in China from 2009. The MIC of colistin for the isolate is 4 μg/ml. Conjugation assays showed that the donor S. flexneri strain has functional and transferable colistin resistance. Sequencing revealed that *mcr-1* was present on a putative composite transposon flanked by inverted repeats of IS*Apl1*.

**IMPORTANCE** There are four species of Shigella, and Shigella flexneri is the most frequently isolated species in low- and middle-income countries (LMICs). In this study, we report a functional, transferable, plasmid-mediated *mcr-1* gene in S. flexneri. We have shown that *mcr-1* is located on a novel composite transposon which is flanked by inverted repeats of IS*Apl1*. The host strain is multidrug resistant, and this multidrug resistance is also transferable. The finding of a functional *mcr-1* gene in S. flexneri, a human-associated Enterobacteriaceae family member, is a cause for concern as infections due to S. flexneri are the main Shigella infections in most low- and middle-income countries.

## INTRODUCTION

Antimicrobial resistance is a major global health issue and is on the national and international agendas of all United Nations member states and many organizations, including the World Health Organization (1). Decreased susceptibility to the most widely used antibiotics, including ampicillin, streptomycin, trimethoprim-sulfamethoxazole, and tetracycline, for enteric pathogens has become a major concern, especially in low- and middle-income countries (LMICs) (2, 3). As a result of the emergence of metallo-beta-lactamases, including NDM-1, and extended-spectrum beta-lactamases such as the CTX-M group in the Enterobacteriaceae, carbapenems and third-generation cephalosporins can no longer be relied upon as treatments for infections caused by multidrug-resistant Enterobacteriaceae (4, 5). For this reason, the polymyxins (colistin and polymyxin B) have become last-resort antibiotics (6) and were reclassified as critically important for human medicine by the WHO in 2011 (7).

Since the first report of transferable, plasmid-mediated colistin resistance conferred by *mcr-1* (8), researchers in different countries have found that many Enterobacteriaceae carry *mcr-1* (9–13). The origins of *mcr-1*-positive strains are varied. Agricultural establishments, retail meat, and patients with infections are three major sources of colistin-resistant bacteria. Since the initial discovery of *mcr-1* in an Escherichia coli isolate from China, the gene has been detected in Southeast Asia, Europe, America, and Africa (14–17). Most of the *mcr-1*-positive strains belong to E. coli, Klebsiella pneumoniae, and Salmonella enterica, while the gene is rarely reported in other Enterobacteriaceae. A recent report described the presence of an *mcr-1*-positive Shigella sonnei strain from Vietnam; however, a colistin resistance phenotype was observed only following transfer to E. coli (18).

Shigella spp. are recognized as etiological agents of diarrhea and have been responsible for serious worldwide epidemics (19). Shigella flexneri is the most frequently isolated species in many countries and is responsible for approximately 10% of all diarrheal episodes in children younger than 5 years (20). S. flexneri 3a is also commonly isolated in male homosexuals in the United States (21) and the United Kingdom (22). Between 2004 and 2015, S. flexneri strains were isolated and collected in China. By screening available isolate collections via PCR, we identified a single *mcr-1*-positive strain of S. flexneri.

## RESULTS

### Bacterial strains and *mcr-1* screening.

A total of 2,127 S. flexneri strains were isolated from samples collected from 13 different areas in China; these were Beijing, Shenyang, Shandong, Henan, Anhui, Hubei, Xinjiang, Gansu, Sichuan, Guizhou, Yunnan, Guangxi, and Guangdong provinces. There are 15 different serotypes among the S. flexneri strains. Most of the strains were isolated from stool samples of patients who were suffering from clinically diagnosed gastroenteritis; a small number of strains (<10%) was isolated from farm and urban environments. Through PCR screening for the presence of *mcr-1* among all the S. flexneri strains, only one *mcr-1*-positive isolate, named C960, was found. The serotype of the positive isolate is Y, and it was isolated from pig stool samples from a pig farm in Guangxi province in 2009.

### Antimicrobial susceptibility and PCR amplification of resistance genes.

Antimicrobial susceptibility tests showed that, in addition to colistin, S. flexneri C960 was resistant to tetracycline, ticarcillin, ampicillin, trimethoprim-sulfamethoxazole, sulfafurazole, and streptomycin (Table 1). Through PCR we found that strain C960 carried other acquired resistance genes, including *qnrS1*, *bla*_TEM-1_, *dfrA14*, and *strB*, which could confer decreased susceptibility to quinolones, beta-lactam antibiotics, trimethoprim, and streptomycin, respectively.

**TABLE 1:** Antimicrobial susceptibility results of S. flexneri C960, E. coli J53, and a transconjugant

Drug	MIC (μg/ml) for the strain^*a*^		
	C960	J53	Transconjugant
Colistin	**4**	≤0.2	**4**
Polymyxin B	**4**	≤0.2	**4**
Tetracycline	**>8**	≤4	**>32**
Ticarcillin	**>64**	≤16	**>64**
Ampicillin	**>16**	≤8	**>32**
Trimethoprim-sulfamethoxazole	**>2**	≤2	**>4**
Sulfafurazole	**>256**	≤16	**>256**
Streptomycin	**>64**	≤2	**>64**
Cefazolin	≤8	≤8	≤8
Cefoxitin	≤8	≤8	≤8
Ceftazidime	≤1	≤1	≤1
Ceftriaxone	≤1	≤1	≤1
Cefoperazone	≤16	≤16	≤16
Ceftiofur	≤0.12	≤0.5	≤0.5
Cefepime	≤8	≤8	≤8
Piperacillin	≤16	≤16	≤16
Amoxicillin-clavulanic acid	≤4	≤4	≤8
Ticarcillin-clavulanic acid	≤16	≤16	≤16
Aztreonam	≤1	≤1	≤1
Imipenem	≤4	≤4	≤4
Nalidixic acid	≤4	≤4	≤8
Ciprofloxacin	≤0.25	≤0.015	≤0.5
Norfloxacin	≤4	≤4	≤4
Levofloxacin	≤2	≤2	≤2
Tobramycin	≤4	≤4	≤4
Gentamicin	≤4	≤4	≤4
Amikacin	≤16	≤16	≤16
Chloramphenicol	≤8	≤8	≤8
Nitrofurantoin	≤32	≤32	≤32
Azithromycin	≤2	≤4	≤4

a Values in boldface indicate resistance; all other values indicate susceptibility.

### Plasmid DNA sequencing and analysis.

**FIG 1:**
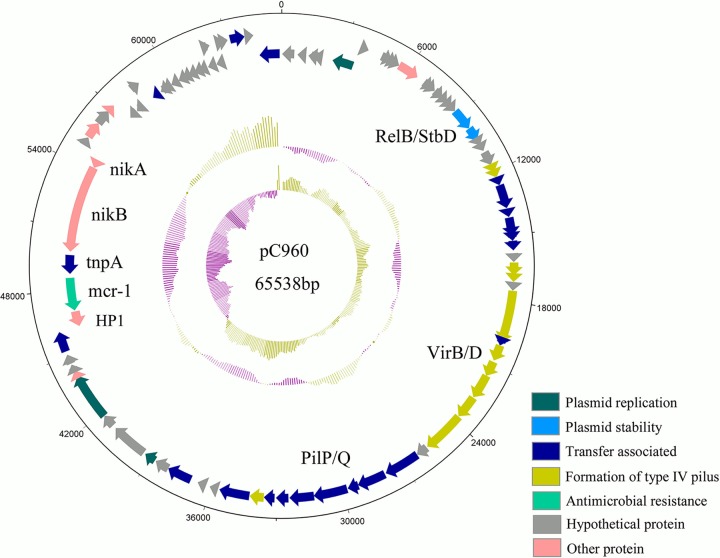
Structure of plasmid pRC960-2 carrying *mcr-1* from Shigella flexneri strain C960. Genes are denoted by arrows and colored based on gene function classification. The innermost circle represents GC content. The second circle presents GC-skew [(G − C)/(G + C)].

**FIG 2:**
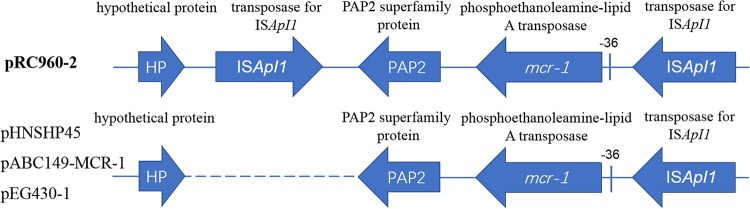
Comparison of the surrounding structure of *mcr-1* in four similar plasmids: pHNSHP45, pABC149-MCR-1, pEG430-1, and pRC960-2. Compared with the sequences of the other three plasmids, an additional, inverted repeat of IS*Apl1* is present downstream of *mcr-1* in plasmid pRC960-2. A single SNP upstream of *mcr-1* (−36) changes from T to C in plasmid pRC960-2.

**FIG 3:**
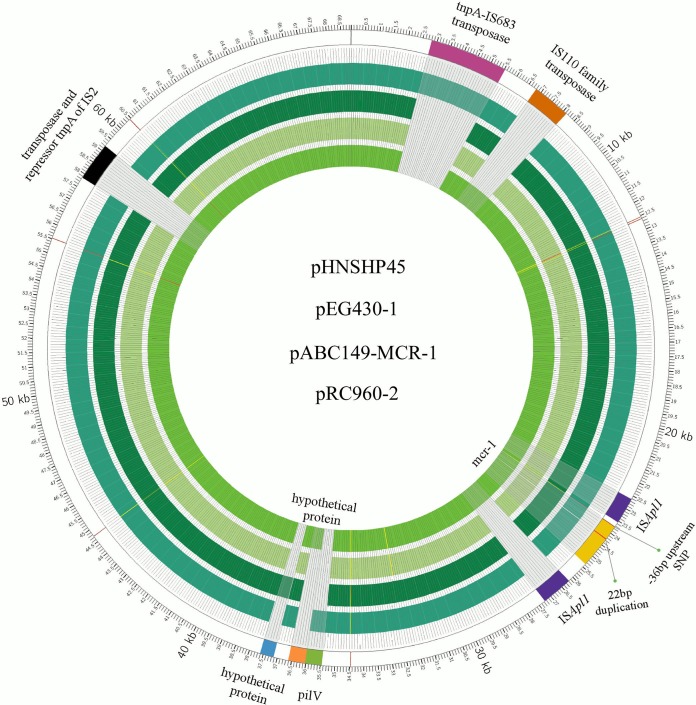
Comparison of the circular genome maps of plasmid sequences of three plasmid genome structures with pHNSHP45 sequence as a reference genome. The green circles represent, from inside to outside, pRC960-2, pEG430-1, pABC149-MCR-1, and pHNSHP45 plasmid sequences, respectively, with respect to the reference pHNSHP45 plasmid genome. In pRC960-2, there is an inverted copy of ISA*pl1* flanking *mcr-1*. A 22-bp duplication of bases 503 to 524 of the *mcr-1* ORF is found in plasmid pEG430-1. The single SNPs upstream of *mcr-1* in pRC960-2, pABC149-MCR-1, and pEG430-1 are the same.

After plasmids of S. flexneri C960 were sequenced and assembled, analysis showed that *mcr-1* in C960 was located on a 65,538-bp plasmid designated pRC960-2. The plasmid has a GC content of 43.2%, contains 92 predicted open reading frames (ORFs), and has a typical IncI2 plasmid backbone (57,756 bp) encoding replication, conjugation apparatus, and stability functions (Fig. 1). The pRC960-2 plasmid sequence (GenBank accession number KY784668) was highly similar (query cover, 95%; identity, 99%) to the sequences of pHNSHP45 (GenBank accession number KP347127) (8) and pABC149-MCR-1 (from E. coli strain ABC149 isolated from the Arabian Peninsula in a clinical blood sample in 2013; GenBank accession number KX013538) (15). Apart from *mcr-1*, there is no other identifiable resistance gene in pRC960-2 (Fig. 1). Compared with the first described *mcr-1* plasmid, pHNSHP45, the region around *mcr-1* in plasmid pRC960-2 had one single nucleotide polymorphism (SNP) in the region upstream of *mcr-1* (Fig. 2). Additionally, there are inverted copies of IS*Apl1* flanking *mcr-1* and some other insertion elements (ISs) in plasmid pRC960-2 compared with the sequences of pHNSHP45 and the other two homologous plasmids (Fig. 2). Except for the inverted repeat of IS*Apl1*, the other genes around *mcr-1* were identical to those in plasmid pABC149-MCR-1, plasmid pEG430-1 (from S. sonnei strain EG430, isolated in a hospital in Vietnam in 2008) (GenBank accession number LT174530) and pHNSHP45 (Fig. 3). Compared with the plasmid pEG430-1, which carries an inactive *mcr-1* in Shigella sonnei, there is no 22-bp duplication in *mcr-1* (in pRC960-2), which has been previously reported to be responsible for inactivity (18). Other detected resistance or resistance-associated genes, including *qnrS1*, *bla*_TEM-1_, *dfrA14*, and *intI1*, were located on a different plasmid without *mcr-1*. This plasmid, pRC960-1, has a length of 75 kb (GenBank accession number KY848295). Based on a BLAST search, plasmid pRC960-1, which contains other resistance genes, aligned closely with the E. coli strain PGRT46 plasmid pPGRT46 found in Nigeria (see Fig. S1 in the supplemental material).

### Conjugation assays.

In order to determine if the plasmids could be transferred, we performed conjugation experiments using S. flexneri C960 with E. coli J53 as the recipient strain. The E. coli J53 transconjugant was found to be resistant to colistin (MIC of 4 mg/liter) to the same extent as the donor. The MIC of other antimicrobials also increased, and the E. coli J53 recipient had almost the same antimicrobial susceptibilities as the donor (Table 1). We detected the *mcr-1*, *qnrS1*, *bla*_TEM-1_, *dfrA14*, and *strB* genes in the transconjugant by PCR. This suggests that both plasmids from S. flexneri C960 transferred into the E. coli J53 recipient and explains the increase in resistance phenotypes observed.

### Excision of Tn*6390*.

**FIG 4:**
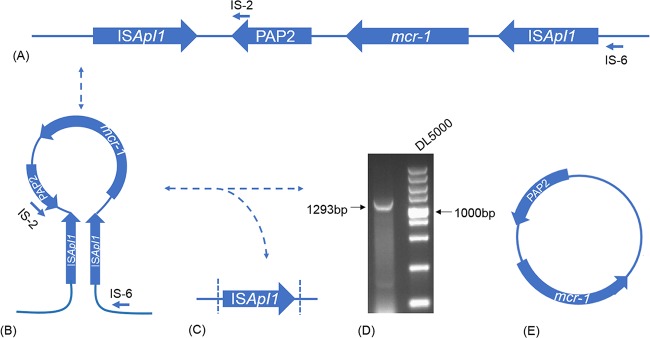
(A) The linear structure of genes surrounding *mcr-1*. The small arrows indicate the locations of the primers used to detect the circular structure. (B) The presumed structure of plasmid pRC960-2 of S. flexneri strain C960. (C) The sequencing result of the PCR products generated by primers IS-2 and IS-6; the fragment amplified by this pair of primers is 1,293 bp and consists of a part of *nikB* and a complete IS*Apl1*. (D) Gel picture of the 1,293-bp PCR products generated by primers IS-2 and IS-6 targeting sequences surrounding Tn*6390*. The amplicon of this pair of primers consists of a part of *nikB* and a complete IS*Apl1*. (E) Schematic representation of the presumed circular structure including *mcr-1*.

We found that there is an inverted copy of IS*Apl1* flanking *mcr-1*, which is unusual as copies of IS*Apl1* are usually directly repeated as in Tn*6330* (23, 24). This putative composite transposon (Tn) was reamplified by PCR, and the PCR products were sequenced to ensure that it was not an artifact due to sequence misassembly of the plasmid reads. The putative composite transposon (IS*Apl1-mcr-1-orf*-IS*Apl1*) was given the designation Tn*6390* by the Transposon Registry (25). We used primers MCR1-RC-F and MCR1-R (19) to test the ability of Tn*6390* to generate a circular intermediate molecule. Through this pair of reverse primers, we obtained a 1,598-bp fragment which contained an intact PAP2 and a part of *mcr-1*. The putative structure of Tn*6390* is shown in Fig. 4B. Then we used primers IS-2 and IS-6 in order to detect the structure formed by two IS*Apl1* elements. The 1,293-bp PCR product (Fig. 4C and D) amplified by IS-2 and IS-6 was the intact IS*Apl1* and a part of *nikB* (located downstream of *mcr-1*). The sequences of PCR products were confirmed by Sanger sequencing.

## DISCUSSION

Among the four species of Shigella, S. flexneri is the most frequently isolated species in LMICs. Humans are the primary reservoir of Shigella spp. (26), which is not the case for Salmonella spp. and E. coli, which are more widely distributed in the environment. Isolation of plasmid-mediated colistin resistance in S. flexneri from animal feces on a farm suggests that it is circulating via the fecal-oral route, at least among the animals on that farm and possibly further afield via the food distribution network. In addition, it suggests that farm environments may be unrecognized reservoirs of S. flexneri.

The use of colistin in Chinese agriculture has been enormous and sustained. Between 2,470 and 2,875 metric tons have been used in the growth of food-producing animals annually in the last 5 years (27). Because of varied and uncontrolled drug administration techniques (injection or addition to feed and water) in food-animal rearing, the selective pressures are high enough to suggest that a large proportion of drug-resistant bacteria emerged from the agricultural sector. This use has allowed for the selection, transfer, and maintenance of plasmid-mediated colistin resistance into clinical strains of E. coli, K. pneumoniae, and Salmonella spp. and rarely into other Enterobacteriaceae. With such sustained selective pressure and transferable resistance circulating among these strains, it is unlikely that this will be the only Shigella flexneri strain found to contain transferable colistin resistance in a farm environment. Also, as only a small number of strains (<10%) were isolated from farm and urban environments, we were surprised to find one with *mcr-1* on a transferable plasmid, which is a relatively high frequency of detection compared to that for clinical strains.

It is of concern that not only colistin resistance was transferred during the filter mating but also a host of mobile elements, including integron, IS, and other resistance genes which are present on the other plasmid were transferred. This suggests that under the selective pressure of colistin, other plasmids conferring multidrug resistance phenotypes can be acquired from the S. flexneri strain. The integron and IS could also help the strain to obtain other resistance elements from the environment. China banned colistin as an animal feed additive recently (28); however, the phenomenon of other inappropriate prophylactic antimicrobial use in farms could still inadvertently select for multiple resistance phenotypes, including colocated colistin resistance.

A novel transposon, Tn*6390*, is found in S. flexneri C960 in which two inverted copies of IS*Apl1* flank *mcr-1*. IS*Apl1* plays a pivotal role in the transposition of *mcr-1* (24, 29); however, almost all other reported structures formed by IS*Apl1-mcr-1-orf*-IS*Apl1* have two direct repeats of IS*Apl1* (23, 24, 30). There is a 1,293-bp PCR product consisting of intact IS*Apl1* and a part of *nikB*, which was presumably the result of a hairpin conformation within the plasmid (Fig. 4B). The consequences for intra- and intercellular mobility of the inverted orientation of IS*Apl1* are under investigation.

Overall, our research shows that a functional and transferable *mcr-1* exists in a multidrug-resistant S. flexneri strain isolated from an agricultural environment. Considering that the *mcr-1* strain was from a small number of agriculturally sourced Shigella strains and that the epidemiology of Shigella sp. infections changes, surveillance of *mcr-1* in both environmental and clinical isolates would be advised.

## MATERIALS AND METHODS

### Strains and *mcr-1* screening.

During the period of 2004 to 2015, a total of 2,127 S. flexneri strains were isolated as part of the national pathogen monitoring system in China. These strains were identified by standard microbiological techniques and then stored in glycerol stocks at −80°C. Colonies were serologically confirmed by slide agglutination with appropriate group-specific polyvalent antiserum, followed by type-specific monovalent antiserum (Denka Seikan, Tokyo, Japan). Basic epidemiological data (date and region of isolation and sex and age of patient) were recorded for each isolate. We retrospectively investigated the presence of *mcr-1* by PCR screening of the historical S. flexneri isolates by using the previously published primers (8) CLR5-F (5′-CGGTCAGTCCGTTTGTTC-3′) and CLR5-R (5′-CTTGGTCGGTCTGTAGGG-3′).

### Antimicrobial susceptibility testing.

The susceptibilities to 28 antimicrobials (ceftazidime, ceftiofur, ceftriaxone, cefepime, cefoperazone, cefazolin, cefoxitin, imipenem, azithromycin, nitrofurantoin, piperacillin, ampicillin, amoxicillin-clavulanic acid, ticarcillin, tetracycline, tobramycin, gentamicin, amikacin, aztreonam, streptomycin, chloramphenicol, ticarcillin-clavulanic acid [Timentin], trimethoprim-sulfamethoxazole, sulfafurazole, nalidixic acid, ciprofloxacin, levofloxacin, and norfloxacin) of the S. flexneri C960 strain, recipient E. coli J53 strain, and the E. coli J53 transconjugants were determined by broth microdilution using a 96-well microtiter plate (Sensititre; Trek Diagnostic Systems, Thermo Fisher Scientific, Inc.). The susceptibilities to colistin and polymyxin B were determined by a microbial viability assay kit using the dye WST (Dojindo Molecular Technologies, Inc., Japan). A reference strain of E. coli (ATCC 25922) was included in the test as a quality control. Interpretation of antimicrobial MICs was performed according to the Clinical and Laboratory Standards Institute criteria (31).

### PCR amplification of resistance genes.

DNA samples were prepared using a TIANamp bacterial DNA kit (Tiangen, Beijing) according to the manufacturer's recommendations. Reactions were performed with 2.5 U of Taq DNA polymerase (TaKaRa, Japan) according to the manufacturer's recommendation. The protocol for the amplification reaction, conducted in a Techne thermocycler (Bio-Red), consisted of initial denaturation at 94°C for 5 min followed by 30 cycles at 94°C for 30 s, 55°C for 30 s, and 72°C for 1 min. A final elongation step was performed at 72°C for 10 min. PCR amplicons were fully sequenced. Other antibiotic resistance determinants were detected by PCR using the primers listed in Table 2. The sequences were analyzed using tools located at the NCBI and aligned to sequences in GenBank.

**TABLE 2:** Primers used in PCR amplification of antibiotic resistance genes

Primer target group and name	Nucleotide sequence (5′ to 3′)	Target	Length (bp)	Reference
Beta-lactamases				
bla_CTX-M-1_ group-F	GGTTAAAAAATCACTGCGTC	*bla*_CTX-M 1_ group	873	This study
bla_CTX-M-1_ group-R	TTACAAACCGTCGGTGACGA	*bla*_CTX-M 1_ group	873	This study
bla_CTX-M-9_ group-F	AGAGTGCAACGGATGATG	*bla*_CTX-M 9_ group	868	This study
bla_CTX-M-9_ group-R	CCAGTTACAGCCCTTCGG	*bla*_CTX-M 9_ group	868	This study
bla_CTX-M-2/8/25_ group-F	ACCGAGCCSACGCTCAA	*bla*_CTX-M-2/8/25_ group	221	This study
bla_CTX-M-2/8/25_ group-R	CCGCTGCCGGTTTTATC	*bla*_CTX-M-2/8/25_ group	221	This study
bla_TEM_-F	ATGAGTATTCAACATTTCCG	*bla*_TEM_	1,080	32
bla_TEM_-R	CCAATGCTTAATCAGTGAGG	*bla*_TEM_	1,080	32
bla_OXA_-F	ATTAAGCCCTTTACCAAACCA	*bla*_OXA_	890	19
bla_OXA_-R	AAGGGTTGGGCGATTTTGCCA	*bla*_OXA_	890	19
bla_VIM_-F3	AGTGGTGAGTATCCGACAG	*bla*_VIM_	509	33
bla_VIM_-R3	ATGAAAGTGCGTGGAGAC	*bla*_VIM_	509	33
bla_NDM-1_-F	GTCTGGCAGCACACTTCCTA	*bla*_NDM-1_	515	This study
bla_NDM-1_-R	TAGTGCTCAGTGTCGGCATC	*bla*_NDM-1_	515	This study
Integrons				
IntI1-F2	ACATGTGATGGCGACGCACGA	*intI1*	569	34
IntI1-R2	ATTTCTGTCCTGGCTGGCGA	*intI1*	569	34
IntI2-F3	CACGGATATGCGACAAAAAGGT	*intI2*	789	34
IntI2-R3	GTAGCAAACGAGTGACGAAATG	*intI2*	789	34
hep58	TCATGGCTTGTTATGACTGT	Class 1 integron variable region	Variable	This study
hep59	GTAGGGCTTATTATGCACGC	Class 1 integron variable region	Variable	This study
hep74	CGGGATCCCGGACGGCATGCACGATTTGTA	Class 2 integron variable region	Variable	19
hep51	GATGCCATCGCAAGTACGAG	Class 2 integron variable region	Variable	19
Chromosomal mutation-mediated quinolone resistance				
gyrA-F	TACACCGGTCAACATTGAGG	*gyrA*	648	35
gyrA-R	TTAATGATTGCCGCCGTCGG	*gyrA*	648	35
gyrB-F	TGAAATGACCCGCCGTAAAGG	*gyrB*	309	35
gyrB-R	GCTGTGATAACGCAGTTTGTCCGGG	*gyrB*	309	35
parC-F	GTACGTGATCATGGACCGTG	*parC*	531	35
parC-R	TTCGGCTGGTCGATTAATGC	*parC*	531	35
parE-F	ATGCGTGCGGCTAAAAAAGTG	*parE*	290	35
parE-R	TCGTCGCTGTCAGGATCGATAC	*parE*	290	35
Plasmid-mediated quinolone resistance				
qnrA-F3	ATTTCTCACGCCAGGATTTG	*qnrA*	516	36
qnrA-R3	GATCGGCAAAGGTYAGGTCA	*qnrA*	516	36
qnrB-F	GATCGTGAAAGCCAGAAAGG	*qnrB*	469	36
qnrB-R	ACGAYGCCTGGTAGTTGTCC	*qnrB*	469	36
qnrD-F	CGAGATCAATTTACGGGGAATA	*qnrD*	656	32
qnrD-R	AACAAGCTGAAGCGCCTG	*qnrD*	656	32
qnrS-F	ACGACATTCGTCAACTGCAA	*qnrS*	417	36
qnrS-R	TAAATTGGCACCCTGTAGGC	*qnrS*	417	36
aac(6′)-Ib-cr-F	GCAACGCAAAAACAAAGTTAGG	*aac(*6′*)-Ib-cr*	560	37
aac(6′)-Ib-cr-R	GTGTTTGAACCATGTACA	*aac(*6′*)-Ib-cr*	560	37

### Plasmid DNA sequencing and analysis.

Plasmid DNA of the S. flexneri C960 strain was extracted using a Qiagen Plasmid Midi kit (Qiagen, Germany). The DNA was used to construct a 600-bp insert library using an NEBNext Ultra II DNA Library Prep kit (NEB, Singapore) and then sequenced by a MiSeq reagent kit, version 3, using the MiSeq platform (Illumina, CA, USA). Raw reads were first assembled into contigs using Newbler, version 3.0, followed by gap filling by local assembly. Pulsed-field gel electrophoresis using S1 nuclease (S1-PFGE) and Southern blotting were used to determine the length of the plasmids. To ensure accuracy, the raw reads were mapped onto the assembled complete genomes to detect misassembly and low-quality regions. In order to get complete plasmid sequences, the gaps were filled through combinatorial PCR and Sanger sequencing on an ABI 3730 sequencer (Life Technologies, CA, USA). The detection and typing of the plasmids were determined using PlasmidFinder (https://cge.cbs.dtu.dk/services/PlasmidFinder/). Each assembled genome was annotated with the Rapid Annotations using Subsystems Technology (RAST) server and verified with the Basic Local Alignment Search Tool (BLAST) against the nonredundant NCBI database (http://blast.ncbi.nlm.nih.gov/Blast.cgi). Annotation of resistance genes, mobile elements, and other genetic structures was based on the relevant databases, including CARD, BacMet, the Beta-Lactamase DataBase (BLDB), and ISfinder. Plasmids pHNSHP45 (GenBank accession number KP347127), pABC149-MCR-1 (GenBank accession number KX013538), pEG430-1 (GenBank accession number LT174530), and pPGRT46 (GenBank accession number KM023153) were used as the reference plasmids for annotation. Plasmid maps were prepared using DNAplotter and Circos. The Tn number was designated by the Transposon Registry (25).

### Conjugation assays.

The ability of *mcr-1* to undergo horizontal gene transfer was assessed by broth and filter mating using a standard E. coli J53 azide-resistant strain as the recipient. The donor/recipient ratio was 10:1, and the temperature was 30°C. MacConkey agar containing 100 mg/liter sodium azide and 2 mg/liter colistin was used to select for E. coli J53 transconjugants. Both Salmonella-Shigella (SS) agar and xylose lysine deoxycholate (XLD) medium (BD Difco, USA) with 2 mg/liter colistin were chosen to select for E. coli J53 transconjugants. Putative transconjugants were confirmed by antimicrobial susceptibility testing and detection of *mcr-1* with PCR and sequencing. No spontaneous resistance to azide could be detected in the S. flexneri donor.

### Detection of the circular structure carrying *mcr-1*.

To test the stability of the Tn*6330*-like structure, primers were designed to detect the circular structure consisting of IS*Apl1-mcr-1-orf*-IS*Apl1* (Table 3). The locations of the primers are shown in Fig. S2 in the supplemental material. The PCR amplicons were fully sequenced.

**TABLE 3:** Primers used in PCR amplification to confirm the arrangement of the transposon IS*Apl1-mcr-1-orf*-IS*Apl1*

Primer name	Nucleotide sequence (5′ to 3′)	Target or function	Length (bp)	Reference
IS-1	TACTTCCTACCGCCATCTTACA	The whole length of Tn*6390*	4,537	This study
IS-4	TACTTCCTACCGACATCTTAC	The whole length of Tn*6390*	4,537	This study
MCR1-RC-F	CTTGGTCGGTCTGTAGGG	To test the ability of Tn*6390* to generate circular intermediate	1,598	23
MCR1-R	TGTCCACGGTTGATGCG	To test the ability of Tn*6390* to generate circular intermediate	1,598	23
IS-5	TCTGTTTGGGGTTGATT	IS*Apl1* and HP1 upstream of *mcr-1*	1,904	This study
IS-7	AAAGTCAAAGACCGCACC	IS*Apl1* and HP1 upstream of *mcr-1*	1,904	This study
IS-2	GAGCCATACGGTGGTGT	The intact IS*Apl1* and a part of *nikB*	1,293	This study
IS-6	CGAATCCGATTTGCTTA	The intact IS*Apl1* and a part of *nikB*	1,293	This study
IS-8	CACAAGAACAAACGGACTGAC	IS*Apl1* downstream *of mcr-1* and a part of *mcr-1*		This study
IS-a	AACGCCTACTGGCTGAGATGAG	To sequence Tn*6390*		This study
IS-b	GGTCGCAACCAGCAAG	To sequence Tn*6390*		This study
IS-c	GTGGCGTTCAGCAGTCATT	To sequence Tn*6390*		This study
IS-d	GCTTACCCACCGAGTAGATT	To sequence Tn*6390*		This study
IS-e	TGGTCGCTGATTGGTTTT	To sequence Tn*6390*		This study
IS-f	GACACCACCGTATGGCTCA	To sequence Tn*6390*		This study

### Accession number(s).

The complete sequences of pRC960-1 and pRC960-2 determined in this study have been deposited in GenBank under the accession numbers KY848295 and KY784668, respectively. All sequencing data from this study are available through the NCBI Sequence Read Archive (SRA) under accession number SRP130733.

## Supplementary Material

Supplemental material
