# Supplementary material for: Transferable Plasmid-Borne mcr-1 in a Colistin-Resistant Shigella flexneri Isolate
Source: Appl Environ Microbiol. 2018 Apr 2;84(8):e02655-17. doi: 10.1128/AEM.02655-17 (PMC5881045; doi:10.1128/AEM.02655-17)
Supplement: Supplemental material [file supp_84_8_e02655-17__index.html]

Transferable Plasmid-Borne mcr-1 in a Colistin-Resistant Shigella flexneri Isolate — Supplemental material 

# Transferable Plasmid-Borne *mcr-1* in a Colistin-Resistant Shigella flexneri Isolate

## Supplemental material

- Supplemental file 1 -

  Structure of plasmid pRC960-1 carrying other resistance genes from *Shigella flexneri* strain C960 and the comparison of plasmid pPGR46 and pRC960-1 (Fig. S1); primers used for sequencing (Fig. S2).

  PDF, 370K
