# Supplementary material for: Transferable Plasmid-Borne mcr-1 in a Colistin-Resistant Shigella flexneri Isolate
Source: Appl Environ Microbiol. 2018 Apr 2;84(8):e02655-17. doi: 10.1128/AEM.02655-17 (PMC5881045; doi:10.1128/AEM.02655-17)
Supplement: Supplemental material [file AEM.02655-17_zam008188453s1.pdf]

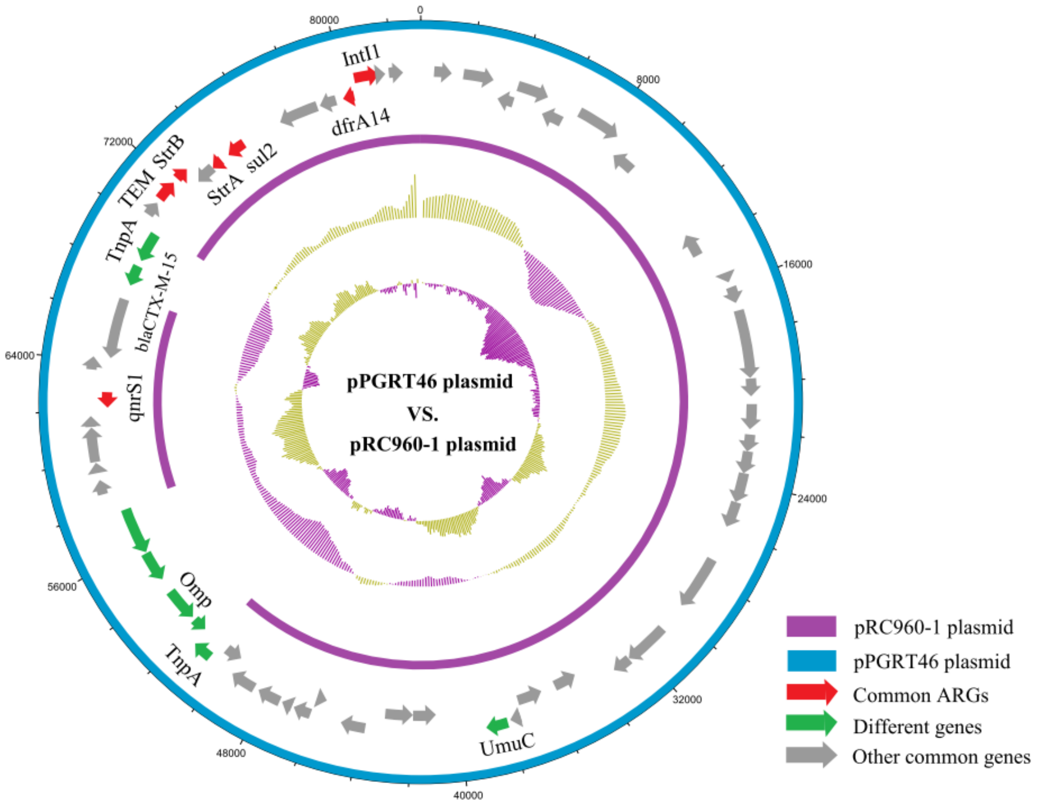

Fig S1

Structure of plasmid pRC960-1 carrying other resistance genes from Shigella flexneri strain C960 and the comparison of plasmid pPGR46 and pRC960-1. Genes are denoted by arrows and colored based on gene function classification. The innermost circle presents GC content. The second circle presents GC-Skew [(G − C)/(G + C)].

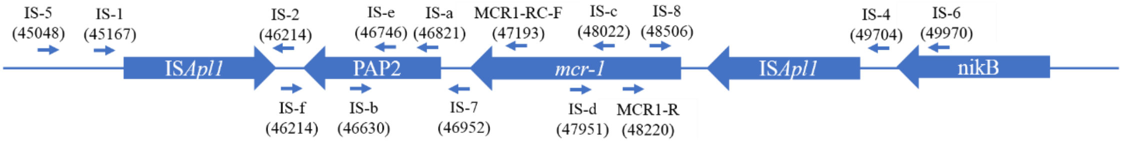

Fig S2

The primers that are used for sequencing. IS-1 and IS-4 are targeting the surrounding sequences of Tn*6390* were utilized to investigate whether the intact Tn*6390* was consistently present among the test bacterial population; IS-5 and IS-7 are targeting the surrounding sequences of IS*Apl1* and PAP2 in the upstream of *mcr-1*; IS-6 and IS-8 are targeting the surrounding sequences of IS*Apl1* in the downstream of *mcr-1*; IS-2 and IS-6 are used to test the structure of Tn*6390*; MCR1-RC-F and MCR1-R are used to test the ability of Tn*6390* to generate circular intermediate; IS-a, IS-b, IS-c, IS-d, IS-e and IS-f are used for sequencing the long fragments. The arrows stand for the direction of the primers, the number in the brackets stand for the locations of the primers in plasmid pRC960-2.
